# Rewiring recovery: Patient-centered neuromodulation interventions for substance use disorders that meet people where they are

**DOI:** 10.1016/j.transm.2025.100179

**Published:** 2025-06-25

**Authors:** Heather Burrell Ward, Sophia H. Blyth, Kristopher Kast

**Affiliations:** Department of Psychiatry and Behavioral Sciences, Vanderbilt University Medical Center, Nashville, TN, United States

**Keywords:** Substance use, Addiction, Treatment protocol, Recovery, Patient-centered care

Substance use disorders (SUDs) affect over 200 million people worldwide (Degenhardt et al., 2018). Existing treatments are effective, although relapse remains all too common (Volkow, 2020; Volkow & Boyle, 2018). Transcranial magnetic stimulation (TMS) has growing interest and evidence as a novel treatment for SUDs (Ekhtiari et al., 2019; Mehta et al., 2023). In 2020, TMS received clearance from the U.S. Food and Drug Administration (FDA) for short-term smoking cessation, marking the first FDA clearance of TMS for a SUD (Zangen et al., 2021).

Over the past two decades, substance use treatment has embraced patient-centered care approaches. Patient-centered care prioritizes an individual’s needs, goals, and preferences as patient and provider engage in shared decision-making (Mead & Bower, 2000; Scholl et al., 2014). A hallmark of patient-centered care involves designing healthcare systems that prioritize patient values to ensure treatment is accessible (Marchand et al., 2019), which improves treatment utilization and substance use outcomes (Davis et al., 2020; Jeansonne et al., 2025; Park et al., 2020; Taylor et al., 2023).

As TMS has been increasingly studied as a novel intervention for SUDs, the principle of patient-centered care has been absent from protocol design (Fig. 1). Although there are many ways to integrate patient-centered care in TMS treatment, we focus on dosing schedule as one example and advocate for patient-centered neuromodulation protocols to support recovery from SUDs. We discuss the benefits and challenges of 1) flexible, 2) accelerated, and 3) extended dosing protocols for SUD treatment.

## Flexible dosing

**Fig. 1.**
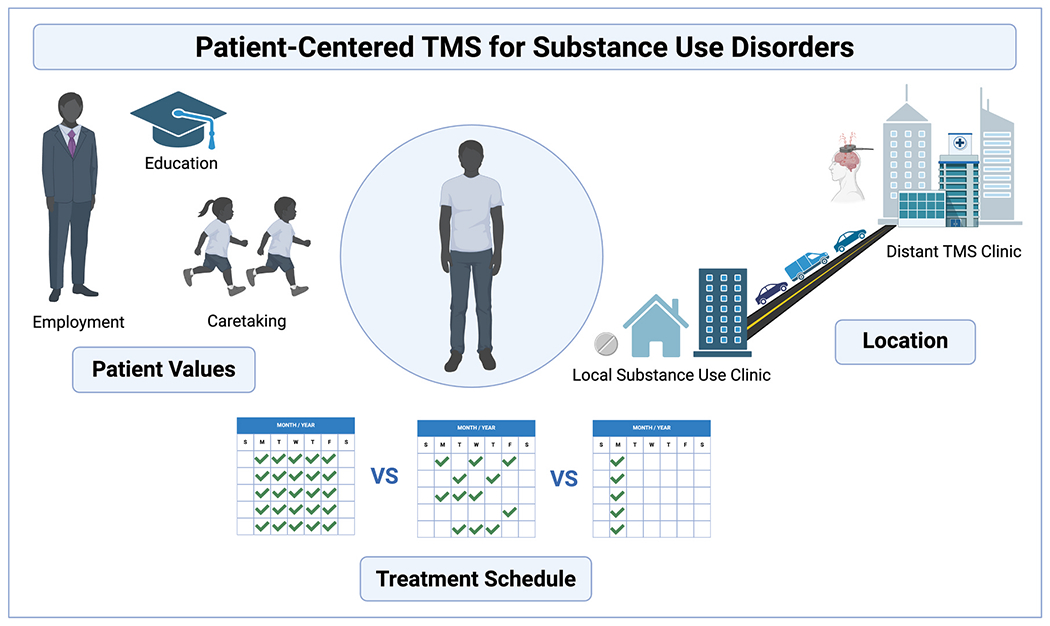
Patient-Centered TMS for Substance Use Disorders. Traditional transcranial magnetic stimulation (TMS) treatment schedules may not be optimal for individuals with substance use disorders (SUDs). To develop patient-centered TMS schedules that promote recovery, protocols should be designed that consider 1) patient values (i.e., employment, education, caretaking); 2) treatment schedule (i.e., daily, flexible, weekly); and 3) location (i.e., co-located TMS and SUD treatment). Created with BioRender.com.

Traditionally, TMS is administered daily for multiple weeks. TMS for major depressive disorder and obsessive-compulsive disorder involves daily treatment for 4–6 weeks (Carmi et al., 2019; O’Reardon et al., 2007). Although each session is brief, these protocols require presenting to an outpatient treatment center daily, which poses challenges for individuals without transportation, who are not located near an TMS treatment center, or who have other educational, occupational, or caretaking responsibilities (Fig. 1). Although these barriers are readily acknowledged in the field, there has been no systematic investigation of barriers to daily TMS treatment. Addressing these barriers may make TMS treatment more accessible for many different populations.

TMS protocols for SUDs have also involved daily treatments for weeks. However, daily treatment may not be feasible for many SUD populations. In a study testing 2 weeks of daily TMS for cannabis use disorder (CUD, n = 9), participants struggled with attendance, and investigators determined daily sessions were infeasible (Sahlem et al., 2020). This is consistent with studies testing daily SUD medication administration. Individuals with opioid use disorder who received weekly (or monthly) depot buprenorphine had higher treatment satisfaction than those who received daily buprenorphine (Lintzeris et al., 2021; Lofwall et al., 2018).

Subsequent TMS trials have tested non-daily dosing, which allows for multiple sessions in one week but with greater flexibility (Addicott et al., 2024; Sahlem et al., 2024). Sahlem et al. tested twice weekly TMS for CUD (n = 72) for 2 weeks (2 sessions/day, 8 total sessions) and observed greater reductions in cannabis use following active TMS compared to sham (Sahlem et al., 2024). Addicott et al. allowed even greater flexibility; in their smoking cessation study (n = 38), participants attended 3–5 visits/week (1–2 sessions/day of intermittent theta burst stimulation, iTBS, to medial prefrontal cortex) across 2–4 weeks until they received 20 sessions (Addicott et al., 2024). Participants from active and sham TMS groups reported reductions in cigarette use, craving, and withdrawal, but there were no significant group differences.

In both studies, participants completed the interventions, and investigators observed reductions in substance use with flexible TMS protocols. These findings are consistent with a study for depression that compared spaced TMS (3 days/week for 6 weeks) to 4 weeks of daily TMS (Galletly et al., 2012). At the end of treatment, the groups were no different, suggesting TMS efficacy is related to total number of treatments and that spacing treatment does not affect response. Although these studies showed that flexible TMS protocols are feasible, improve retention, and may reduce substance use, both studies failed to achieve their primary endpoints. Notably, these two studies were testing 1) a novel protocol and target (iTBS to medial prefrontal cortex for nicotine use (Addicott et al., 2024)) or 2) a novel indication (CUD (Sahlem et al., 2024)) for which it is unknown if even *daily* treatment is effective. The current literature on flexible dosing is sparse, and research is needed to directly test if flexible dosing is as effective as daily treatment.

## Accelerated dosing

Daily TMS sessions pose challenges even for depression treatment. Newer “accelerated” TMS protocols apply multiple TMS sessions/day to condense treatment course and achieve faster time to response (van Rooij et al., 2024). A recent study testing accelerated TMS for depression observed rapid reductions in depressive symptoms, which generated excitement in the neuromodulation community (Cole et al., 2022). As a result, there has been enthusiasm for accelerated protocols for psychiatric and SUDs. Interestingly, a naturalistic study of accelerated deep TMS found that the efficacy of multiple sessions/day was comparable to daily treatment (Roth et al., 2023), suggesting an evidence-based, daily TMS protocol for SUDs could be scaled to multiple treatments/day without compromising efficacy. Although few studies have tested accelerated TMS protocols for SUDs, there have been promising results. Steele and colleagues tested 3 iTBS sessions/day for 10 days (30 sessions) for cocaine use disorder and observed significant reductions in cocaine use (Steele et al., 2019). However, there are concerns about the feasibility and effectiveness of accelerated protocols in a SUD population.

As TMS is being investigated as a novel treatment for SUDs, patient-centered protocols should be developed that integrate with existing treatment and support recovery from SUDs.

The American Society of Addiction Medicine defines recovery as “the process of sustained action that addresses the biological, psychological, social, and spiritual disturbance inherent in addiction” (ASAM, 2018). Beyond the acute period of detoxification and withdrawal, recovery includes social and occupational recovery – reestablishing connections with family and friends and sustaining employment.

Therefore, rigid or intensive TMS treatment protocols run counter to recovery goals. Daily treatment, in addition to being logistically challenging, may also interfere with recovery goals, such as participating in childcare or obtaining and maintaining gainful employment (Fig. 1). Accelerated protocols likely face the same challenges. The current FDA-cleared accelerated protocol for depression involves 10 sessions per day (i.e., 10 h) for 5 consecutive days (Cole et al., 2022), which poses an obvious challenge to someone working a standard full-time job who may have limited flexibility in their schedule.

## Extended dosing

Accelerated protocols for depression were developed to shorten time to response. However, it is well-accepted by clinicians and patients alike that recovery from substance use requires time and behavior change. Accelerated TMS protocols that provide treatment over several days may not be optimal for neural and behavior change. Extended treatment may also allow TMS to treat persistent symptoms of addiction. Even after medically supervised withdrawal, symptoms of post-acute withdrawal can persist for months. Post-acute withdrawal symptoms include anxiety, fatigue, insomnia, and mood disturbance and have been observed in opioid, alcohol, stimulant, and cannabis use disorders. Therefore, longer TMS treatment protocols could treat persistent post-acute withdrawal symptoms.

Alternative TMS protocols should be designed to integrate into ongoing systems of care and support SUD recovery (Fig. 1). One solution is weekly accelerated dosing, where patients receive weekly treatment that consists of multiple TMS sessions coupled with psychotherapy. The timing of TMS could be easily scheduled to coincide with clinical care, such as buprenorphine treatment visits.

Weekly TMS treatment is consistent with standard substance use treatment, which often occurs weekly. For example, following initiation of buprenorphine for opioid use disorder, patients may present to Bridge Clinics for weekly medication management, psychotherapy, peer support, and other medical care (Marcovitz et al., 2024). Moreover, psychotherapy for substance use or other psychiatric disorders is generally delivered weekly. Standard smoking cessation clinical trials frequently involve weekly visits that include psychotherapy to support abstinence and relapse prevention (Anthenelli et al., 2016).

Relapse prevention refers to the period after acute abstinence when an individual sustains abstinence and recovery for a longer period of time. More flexible TMS protocols can be used for relapse prevention. Several TMS protocols (including the FDA-cleared smoking cessation protocol) involve several weeks of daily treatment followed by single TMS sessions delivered weekly for relapse prevention (Zangen et al., 2021). However, the effectiveness of TMS for relapse prevention has not been tested. Weekly accelerated treatment could be spaced out (i.e., biweekly, monthly) and tested for relapse prevention. As an individual progresses from early to sustained abstinence, the most effective neuromodulation targets may shift, e.g., from craving to inhibitory control or management of negative affect. Therefore, different TMS targets and schedules may be necessary at various stages of recovery and should be tested in future research.

## Conclusion

TMS has great potential as a novel intervention for SUDs. In contrast to other interventions, TMS has flexibility in its administration schedule and represents a unique opportunity to design treatments that are patient-centered and support recovery from substance use. TMS dosing schedules can be tailored to a patient’s values and needs. There are a variety of dosing schedules (i.e., flexible, accelerated, extended) that should be first tested for efficacy (i.e., are multiple accelerated TMS sessions delivered in one day as effective as daily or non-daily treatment?) then feasibility. Rather than applying one-size-fits-all treatment schedules, patients could select a treatment schedule that is feasible given their values and resources but does not compromise effectiveness. To achieve this goal, funding agencies should call for proposals testing alternative TMS schedules for SUDs so that TMS is accessible, feasible, and highly effective for the patients who need it most.

## References

[R1] Addicott MA, Kinney KR, Saldana S, Ip EH, DeMaioNewton H, Bickel WK, Hanlon CA. A randomized controlled trial of intermittent theta burst stimulation to the medial prefrontal cortex for tobacco use disorder: Clinical efficacy and safety. Drug Alcohol Depend. 2024;258, 111278. 10.1016/j.drugalcdep.2024.111278. 38579605 PMC11088513

[R2] Anthenelli RM, Benowitz NL, West R, St Aubin L, McRae T, Lawrence D, Ascher J, Russ C, Krishen A, Evins AE. Neuropsychiatric safety and efficacy of varenicline, bupropion, and nicotine patch in smokers with and without psychiatric disorders (EAGLES): a double-blind, randomised, placebo-controlled clinical trial. Lancet. 2016;387(10037):2507–2520. 10.1016/s0140-6736(16)30272-0. 27116918

[R3] Carmi L, Tendler A, Bystritsky A, Hollander E, Blumberger DM, Daskalakis J, Ward H, Lapidus K, Goodman W, Casuto L, Feifel D, Barnea-Ygael N, Roth Y, Zangen A, Zohar J. Efficacy and safety of deep transcranial magnetic stimulation for obsessive-compulsive disorder: A prospective multicenter randomized double-blind placebo-controlled trial. American Journal of Psychiatry. 2019;176(11):931–938. 10.1176/appi.ajp.2019.18101180. 31109199

[R4] Cole EJ, Phillips AL, Bentzley BS, Stimpson KH, Nejad R, Barmak F, Veerapal C, Khan N, Cherian K, Felber E, Brown R, Choi E, King S, Pankow H, Bishop JH, Azeez A, Coetzee J, Rapier R, Odenwald N, Williams NR. Stanford neuromodulation therapy (SNT): A double-blind randomized controlled trial. American Journal of Psychiatry. 2022;179(2):132–141. 10.1176/appi.ajp.2021.20101429. 34711062

[R5] Degenhardt L, Charlson F, Ferrari A, Santomauro D, Erskine H, Mantilla-Herrara A, Whiteford H, Leung J, Naghavi M, Griswold M, Rehm J, Hall W, Sartorius B, Scott J, Vollset SE, Knudsen AK, Haro JM, Patton G, Kopec J, …, Vos T. The global burden of disease attributable to alcohol and drug use in 195 countries and territories, 1990–2016: a systematic analysis for the Global Burden of Disease Study 2016. Lancet Psychiatry. 2018;5(12):987–1012. 10.1016/s2215-0366(18)30337-7. 30392731 PMC6251968

[R6] Davis EL, Kelly PJ, Deane FP, Baker AL, Buckingham M, Degan T, Adams S. The relationship between patient-centered care and outcomes in specialist drug and alcohol treatment: A systematic literature review. Substance Abuse. 2020;41(2):216–231. 10.1080/08897077.2019.1671940. 31638870

[R7] Ekhtiari H, Tavakoli H, Addolorato G, Baeken C, Bonci A, Campanella S, Castelo-Branco L, Challet-Bouju G, Clark VP, Claus E, Dannon PN, Del Felice A, den Uyl T, Diana M, di Giannantonio M, Fedota JR, Fitzgerald P, Gallimberti L, Grall-Bronnec M, …, Hanlon CA. Transcranial electrical and magnetic stimulation (tES and TMS) for addiction medicine: A consensus paper on the present state of the science and the road ahead. Neuroscience Biobehaviour Review. 2019. 10.1016/j.neubiorev.2019.06.007. PMC7293143 31271802

[R8] Galletly C, Gill S, Clarke P, Burton C, Fitzgerald PB. A randomized trial comparing repetitive transcranial magnetic stimulation given 3 days/week and 5 days/week for the treatment of major depression: is efficacy related to the duration of treatment or the number of treatments? Psychological Medicine. 2012;42(5):981–988. 10.1017/S0033291711001760. 21910937

[R9] Jeansonne S, Dopp AR, Phillips C, Cook M, Brown L, Komaromy M, Page K, Watkins KE. Implementing a patient-centered, rapid-access substance use treatment pathway in primary care. Psychiatric Services. 2025, appips20240150. 10.1176/appi.ps.20240150. 39818995 PMC12520321

[R10] Lintzeris N, Dunlop AJ, Haber PS, Lubman DI, Graham R, Hutchinson S, Arunogiri S, Hayes V, Hjelmström P, Svedberg A, Peterson S, Tiberg F. Patient-reported outcomes of treatment of opioid dependence with weekly and monthly subcutaneous depot vs daily sublingual buprenorphine: A randomized clinical trial. JAMA Network Open. 2021;4(5), e219041. 10.1001/jamanetworkopen.2021.9041. 33970256 PMC8111483

[R11] Lofwall MR, Walsh SL, Nunes EV, Bailey GL, Sigmon SC, Kampman KM, Frost M, Tiberg F, Linden M, Sheldon B, Oosman S, Peterson S, Chen M, Kim S. Weekly and monthly subcutaneous buprenorphine depot formulations vs daily sublingual buprenorphine with naloxone for treatment of opioid use disorder: A randomized clinical trial. JAMA Internal Medicine. 2018;178(6):764–773. 10.1001/jamainternmed.2018.1052. 29799968 PMC6145749

[R12] Marchand K, Beaumont S, Westfall J, MacDonald S, Harrison S, Marsh DC, Schechter MT, Oviedo-Joekes E. Conceptualizing patient-centered care for substance use disorder treatment: findings from a systematic scoping review. Substance Abuse Treatment, Prevention, and Policy. 2019;14(1):37. 10.1186/s13011-019-0227-0. 31511016 PMC6739978

[R13] Marcovitz D, Dear ML, Donald R, Edwards DA, Kast KA, Le TDV, Shah MV, Ferrell J, Gatto C, Hennessy C, Buie R, Rice TW, Sullivan W, White KD, Van Winkle G, Wolf R, Lindsell CJ, Investigators, V. L. H. S. P. Effect of a co-located bridging recovery initiative on hospital length of stay among patients with opioid use disorder: the bridge randomized clinical trial. JAMA Network Open. 2024;7(2), e2356430. 10.1001/jamanetworkopen.2023.56430. 38411964 PMC10900965

[R14] Mead N, Bower P. Patient-centredness: a conceptual framework and review of the empirical literature. Social Science & Medicine. 2000;51(7):1087–1110. 10.1016/s0277-9536(00)00098-8. 11005395

[R15] American Society of Addiction Medicine (ASAM) (2018). Public Policy Statement on the Role of Recovery in Addiction Care.

[R16] Mehta DD, Praecht A, Ward HB, Sanches M, Sorkhou M, Tang VM, Steele VR, Hanlon CA, George TP. A systematic review and meta-analysis of neuromodulation therapies for substance use disorders. Neuropsychopharmacology. 2023. 10.1038/s41386-023-01776-0. PMC10876556 38086901

[R17] O’Reardon JP, Solvason HB, Janicak PG, Sampson S, Isenberg KE, Nahas Z, McDonald WM, Avery D, Fitzgerald PB, Loo C, Demitrack MA, George MS, Sackeim HA. Efficacy and safety of transcranial magnetic stimulation in the acute treatment of major depression: a multisite randomized controlled trial. Biological Psychiatry. 2007;62(11):1208–1216. 10.1016/j.biopsych.2007.01.018. 17573044

[R18] Park SE, Mosley JE, Grogan CM, Pollack HA, Humphreys K, D’Aunno T, Friedmann PD. Patient-centered care’s relationship with substance use disorder treatment utilization. Journal of Substance Abuse Treat. 2020;118, 108125. 10.1016/j.jsat.2020.108125. PMC7528396 32972650

[R19] Roth Y, Hanlon CA, Pell G, Zibman S, Harmelech T, Muir OS, MacMillan C, Prestley T, Purselle DC, Knightly T, Tendler A. Real world efficacy and safety of various accelerated deep TMS protocols for major depression. Psychiatry Research. 2023;328, 115482. 10.1016/j.psychres.2023.115482. 37738684

[R20] Sahlem GL, Caruso MA, Short EB, Fox JB, Sherman BJ, Manett AJ, Malcolm RJ, George MS, McRae-Clark AL. A case series exploring the effect of twenty sessions of repetitive transcranial magnetic stimulation (rTMS) on cannabis use and craving. Brain Stimulation. 2020;13(1):265–266. 10.1016/j.brs.2019.09.014. 31619347 PMC7263465

[R21] Sahlem GL, Kim B, Baker NL, Wong BL, Caruso MA, Campbell LA, Kaloani I, Sherman BJ, Ford TJ, Musleh AH, Kim JP, Williams NR, Manett AJ, Kratter IH, Short EB, Killeen TK, George MS, McRae-Clark AL. A preliminary randomized controlled trial of repetitive transcranial magnetic stimulation applied to the left dorsolateral prefrontal cortex in treatment seeking participants with cannabis use disorder. Drug Alcohol Depend. 2024;254, 111035. 10.1016/j.drugalcdep.2023.111035. 38043228 PMC10837319

[R22] Scholl I, Zill JM, Härter M, Dirmaier J. An integrative model of patient-centeredness - a systematic review and concept analysis. PLoS One. 2014;9(9), e107828. 10.1371/journal.pone.0107828. 25229640 PMC4168256

[R23] Steele VR, Maxwell AM, Ross TJ, Stein EA, Salmeron BJ. Accelerated intermittent theta-burst stimulation as a treatment for cocaine use disorder: A proof-of-concept study. Frontier Neuroscience. 2019;13:1147. 10.3389/fnins.2019.01147. PMC6831547 31736689

[R24] Taylor JL, Wakeman SE, Walley AY, Kehoe LG. Substance use disorder bridge clinics: models, evidence, and future directions. Addiction Science & Clinical Practice. 2023;18(1):23. 10.1186/s13722-023-00365-2. 37055851 PMC10101823

[R25] van Rooij SJH, Arulpragasam AR, McDonald WM, Philip NS. Accelerated TMS - moving quickly into the future of depression treatment. Neuropsychopharmacology. 2024;49(1):128–137. 10.1038/s41386-023-01599-z. 37217771 PMC10700378

[R26] Volkow ND. Personalizing the treatment of substance use disorders. American Journal of Psychiatry. 2020;177(2):113–116. 10.1176/appi.ajp.2019.19121284. 32008390

[R27] Volkow ND, Boyle M. Neuroscience of addiction: Relevance to prevention and treatment. American Journal of Psychiatry. 2018;175(8):729–740. 10.1176/appi.ajp.2018.17101174. 29690790

[R28] Zangen A, Moshe H, Martinez D, Barnea-Ygael N, Vapnik T, Bystritsky A, Duffy W, Toder D, Casuto L, Grosz ML, Nunes EV, Ward H, Tendler A, Feifel D, Morales O, Roth Y, Iosifescu DV, Winston J, Wirecki T, …, George MS. Repetitive transcranial magnetic stimulation for smoking cessation: A pivotal multicenter double-blind randomized controlled trial. World Psychiatry. 2021;20(3):397–404. 10.1002/wps.20905. 34505368 PMC8429333

